# MRTF-A controls myofibroblastic differentiation of human multipotent stromal cells and their tumour-supporting function in xenograft models

**DOI:** 10.1038/s41598-019-48142-z

**Published:** 2019-08-13

**Authors:** Sara Werner, Jana Lützkendorf, Thomas Müller, Lutz P. Müller, Guido Posern

**Affiliations:** 1 Institute for Physiological Chemistry, Medical Faculty, Martin Luther University Halle-Wittenberg, Hollystrasse 1, 06114 Halle (Saale), Germany; 2 University Clinic of Internal Medicine IV, Medical Faculty, Martin Luther University Halle-Wittenberg, Ernst-Grube-Strasse 40, 06120 Halle (Saale), Germany

**Keywords:** Molecular medicine, Cell signalling, Cancer microenvironment, Molecular medicine, Cell signalling

## Abstract

Tumour growth and metastatic colonization is strongly influenced by the tumour stroma, including cancer-associated fibroblasts (CAF). Multipotent mesenchymal stromal cells (MSC) are a possible source of CAF following myofibroblastic differentiation, and we have previously shown that MSC support tumour growth. Triggered by tumour cell-derived factors like transforming growth factor β1 (TGF-β1), myofibroblastic MSC differentiation is associated with the increased expression of markers including alpha smooth muscle actin (α-SMA). Here we show that myocardin-related transcription factor A (MRTF-A) plays an important role in myofibroblastic differentiation of primary human MSC *in vitro* and their tumour-supporting function *in vivo*. Recombinant TGF-β1 or tumour cell conditioned medium (TCM) elevated α-SMA, calponin 1 and collagen 1 A1 (COL1A1) amount on mRNA and protein level in MSC. This correlated with increased MRTF-A activity during MSC differentiation. MRTF-A knockdown by siRNA or shRNA impaired TGF-β1 and TCM induction of α-SMA and calponin 1, but not of COL1A1. Mixed xenograft experiments using HCT8 colorectal carcinoma cells and primary MSC of different donors revealed a significant reduction in tumour weight and volume upon MRTF-A knockdown in MSC. Our study suggests that MRTF-A is involved in the functional differentiation of MSC towards a tumour-promoting CAF phenotype *in vivo*.

## Introduction

The tumour microenvironment plays an essential role in the pathophysiology of tumours and comprises extracellular matrix (ECM) and non-malignant cells. Tumour formation and metastatic colonization depends on intricate crosstalk between cancer and stromal cells. Within the tumour stroma, CAF facilitate important tumour-supporting functions. Differentiation towards CAF resembles the wound healing response by activated fibroblast, resulting in the desmoplastic reaction within tumours [reviewed by^1^]. CAF are myofibroblast-like cells characterized by an elevated expression of various cytoskeletal and matrix components including α-SMA, collagens and calponin 1^2,3^. Several studies suggest that CAF are derived from infiltrating or resident MSC^4^. MSC reside throughout life as pericytes in many tissues, can be easily isolated and are defined by standard criteria, including adherence to plastic, a defined immune phenotype and multi-lineage differentiation ability^5,6^. Their location as perivascular cells suggest a possible role for MSC in initial cell-to-cell contacts with circulating tumour cells thereby providing a pre-metastatic niche. Moreover, MSC actively migrate towards tumours^7^. Tumour-integrated MSC with myofibroblast characteristics enhance tumour growth in colorectal carcinoma (CRC) xenograft models^8,9^. We previously demonstrated that the MSC-mediated growth support of CRC xenografts depends on expression of ECM components and integrins^9^.

Myofibroblastic differentiation is linked to TGF-β1 signalling and the MRTF-A. The activity of MRTF-A is regulated by Rho family GTPases and depends on G-actin which forms repressive complexes with MRTF-A^10^. Upon activation by altered actin dynamics and mechanical stimulation, MRTF-A is released and induces SRF (serum response factor)-dependent transcription in the nucleus [reviewed by^11,12^]. Actin- and MRTF-regulated genes include a plethora of cytoskeletal and cell adhesion components, including α-SMA, calponin 1 and collagen 1^13,14^. Genome-wide expression studies in murine models of CAF revealed a significant overlap with TGF-β1- and Rho-Actin-MRTF signatures^14–16^. Although the details of the pathway crosstalk are elusive, MRTF-A is implicated in TGF-β1 mediated myofibroblastic differentiation in various cell systems^17,18^. MRTF-A-mediated induction of integrins, actomyosin and septin components alters fibroblast motility, contractility and matrix remodelling^19–21^. In immortalized murine CAF, MRTF-A was recently shown to be required for collagen invasion of 4T1 tumour cells and gel contraction^21^.

Here we investigate the role of MRTF-A for myofibroblastic differentiation of MSC and their tumour-supporting capability. Primary MSC isolated from human bone marrow were differentiated by TGF-β1 and tumour-cell conditioned medium to myofibroblasts with induced expression of the differentiation markers α-SMA, calponin 1 and COL1A1. We found that MRTF-A is activated during myofibroblast differentiation and required for α-SMA and calponin expression, but not for COL1A1 induction. Using lentiviral infection, stable knockdown of MRTF-A impaired myofibroblast marker expression in MSC of various donors. In mixed xenograft experiments, MSC partially depleted for MRTF-A showed a reduced tumour-supporting effect towards HCT8 colorectal carcinoma cells *in vivo*. The results show that MRTF-A controls myofibroblastic differentiation and CAF functions in primary human MSC.

## Results

### Myofibroblastic differentiation of primary human MSC

**Figure 1:**
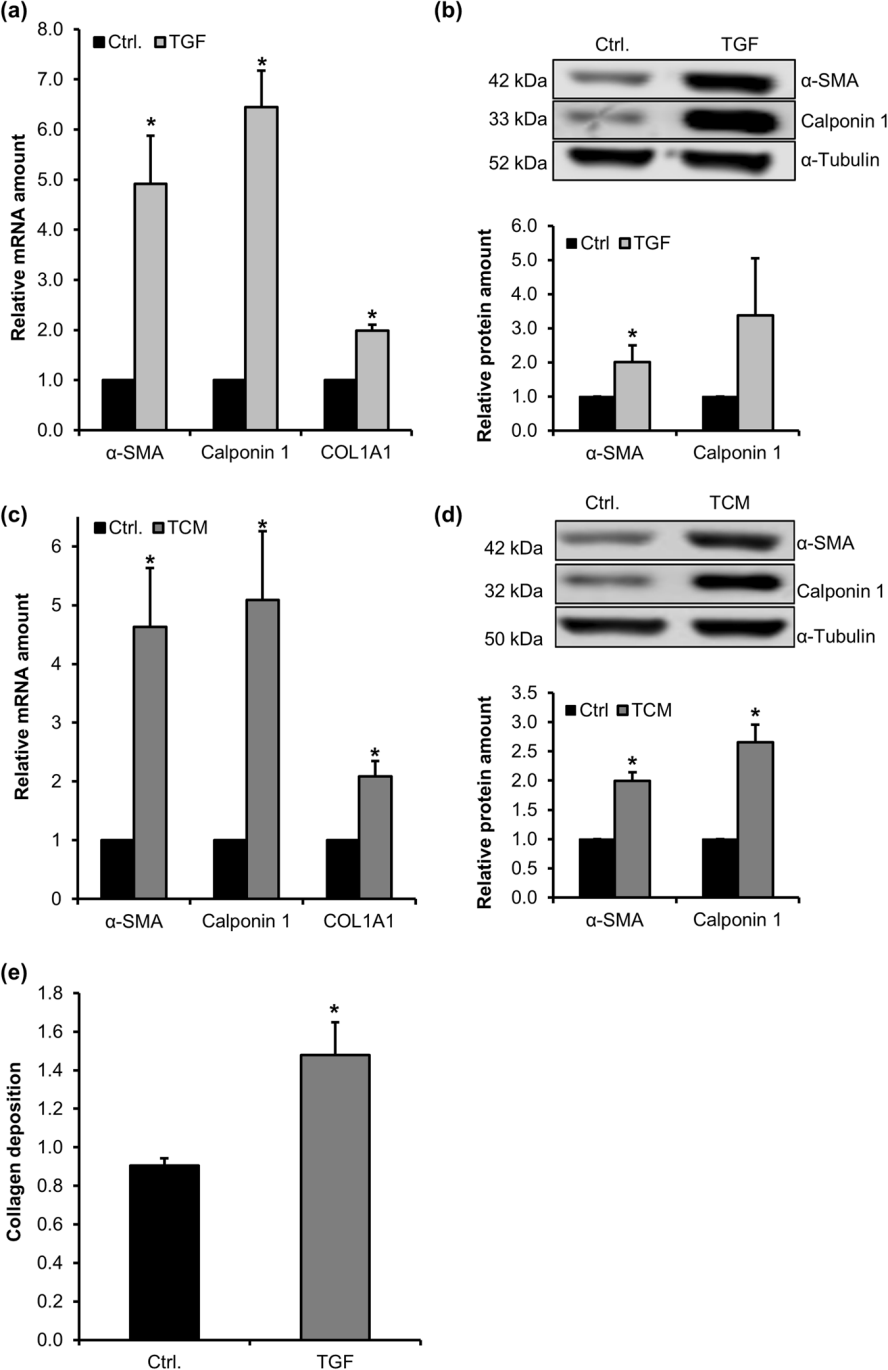
TGF-β1 and TCM induce expression of myofibroblastic markers in MSC. Bone-marrow derived MSC were isolated from the iliac crest of individual patients and treated with TGF-β1 (TGF, 10 ng/ml) **(a)** or tumour cell conditioned medium (TCM) **(c)** for 24 h. Relative mRNA of calponin 1, α-SMA and COL1A1 was quantified by real-time RT-PCR and normalized to ALAS and GAPDH. Western blots of α-SMA and calponin 1 in MSC after 48 h of TGF-β1 (TGF) **(b)** or TCM **(d)** treatment. Relative protein amounts were normalized to the α-tubulin signal. All data were normalized to the value for the starved control (Ctrl.) which was set to 1. **(e)** Relative collagen deposition determined by Sirius Red staining normalized to cell accumulation following treatment with TGF-β1 for 96 h. Error bars correspond to SD (n = 3). Asterisks indicate significant differences *p ≤ 0.05 according to an unpaired Student’s t test.

We have previously observed a tumour-promoting activity of MSC in a CRC xenograft model^9^. To investigate whether this activity involves a myofibroblastic differentiation, we firstly isolated primary human MSC and subjected them to treatment with recombinant TGF-β1 for 24 h. This resulted in a significant upregulation of the expression of α-SMA (ACTA2), calponin 1 (CNN1) and collagen (COL1A1) genes in cells of 3 individual donors (Fig. 1a). Furthermore, a profound increase of α-SMA and calponin 1 was detected at protein level following TGF-β1 treatment (Fig. 1b). MSC differentiation into CAF is thought to require signals from the tumour cells. We thus tested tumour cell conditioned medium (TCM) from the CRC cell line HCT8 for which we reported a promotion of xenograft growth by MSC^9^. When MSC were treated with control medium, TGF-β1 or TCM, experiments were done under serum-starved conditions. Similar to the results obtained with TGF-β1 treatment, the mRNA of α-SMA, calponin 1 and COL1A1 were significantly induced in MSC by TCM treatment (Fig. 1c). On protein level, α-SMA and calponin 1 increased in lysates of TCM-stimulated MSC (Fig. 1d and Suppl. Fig. S1). Moreover, collagen deposition was elevated by around 50% following 96 h of TGF-β1 stimulation, as determined by normalized Sirius Red staining (Fig. 1e).

**Figure 2:**
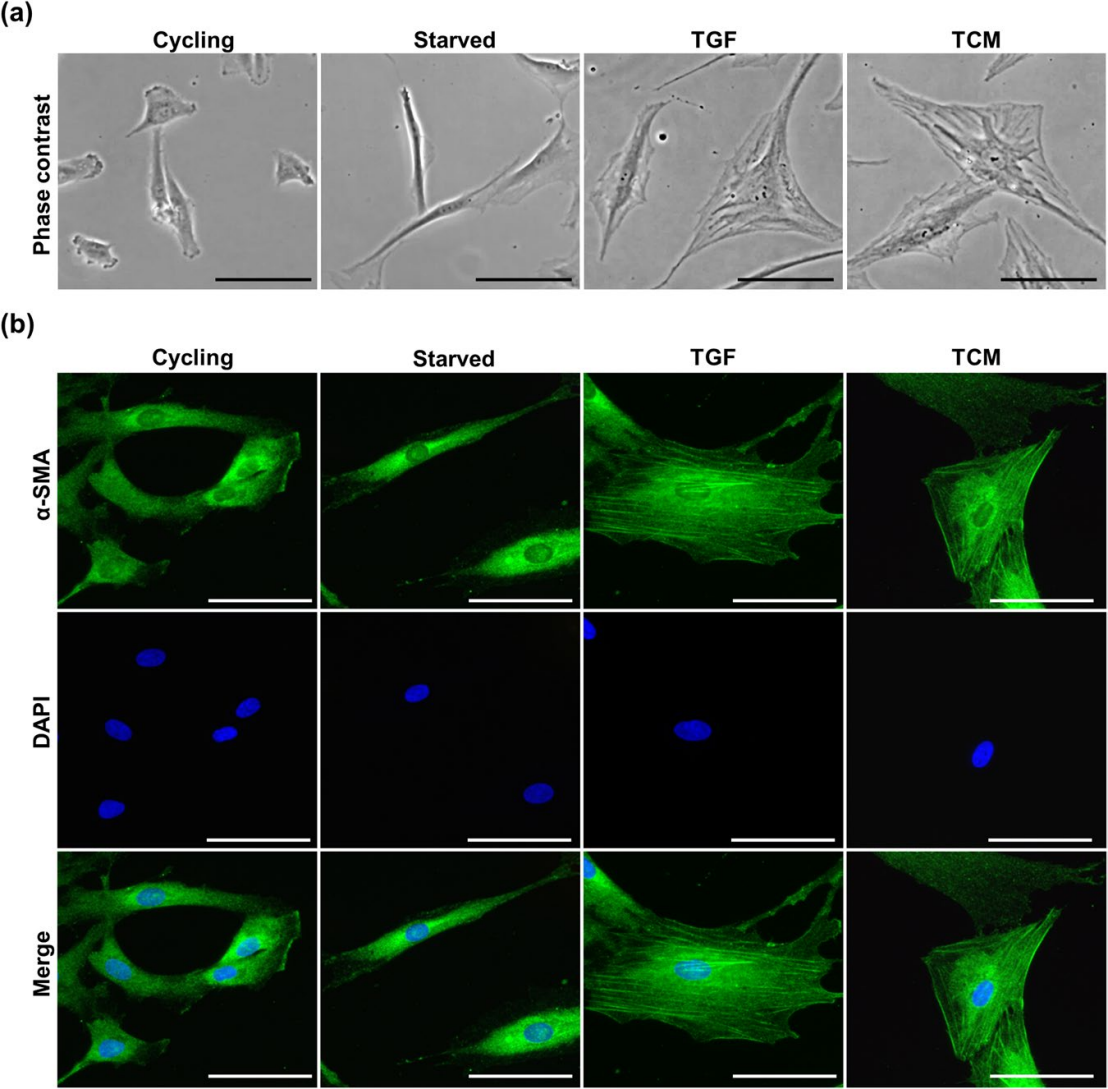
TGF-β1 and TCM cause morphological and cytoskeletal changes in MSC. **(a)** Phase contrast images of the morphology of untreated proliferating MSC (cycling), quiescent MSC (starved) and MSC differentiated by TGF-β1 or TCM for 48 h. **(b)** Immunofluorescence images of α-SMA in MSC. Cycling, starved and treated (TGF, TCM) MSC were stained with anti-α-SMA and DAPI after 48 h of treatment. 20 x magnification, scale bars 20 µm.

MSC differentiation is often associated with changes of the cellular shape. We observed a flattened cell shape with a profound spread-out morphology upon both TGF-β1 and TCM treatment (Fig. 2a). In contrast, starved cells lacking recombinant TGF-β1 or HCT8 secreted factors were characterized by spindle-shaped narrow cell bodies. Cycling cells, grown in medium supplemented with 10% human platelet lysate, were also smaller but more circular, sometimes exhibiting lamellipodia-like protrusions. Moreover, immunofluorescence staining revealed a change of α-SMA expression pattern and intensity (Fig. 2b). Distinct fibres were visible in the differentiated cells compared to the control (starved) and cycling cells and a weak α-SMA signal was diffusely localized throughout the cytoplasm. Together, these data suggest that human primary MSC are differentiated by TGF-β1 and HCT8-conditioned medium into cells with myofibroblast-like characteristics.

### Tumour cell-conditioned medium contains TGF-β1 and activates Alk5/SMAD2 signalling

**Figure 3:**
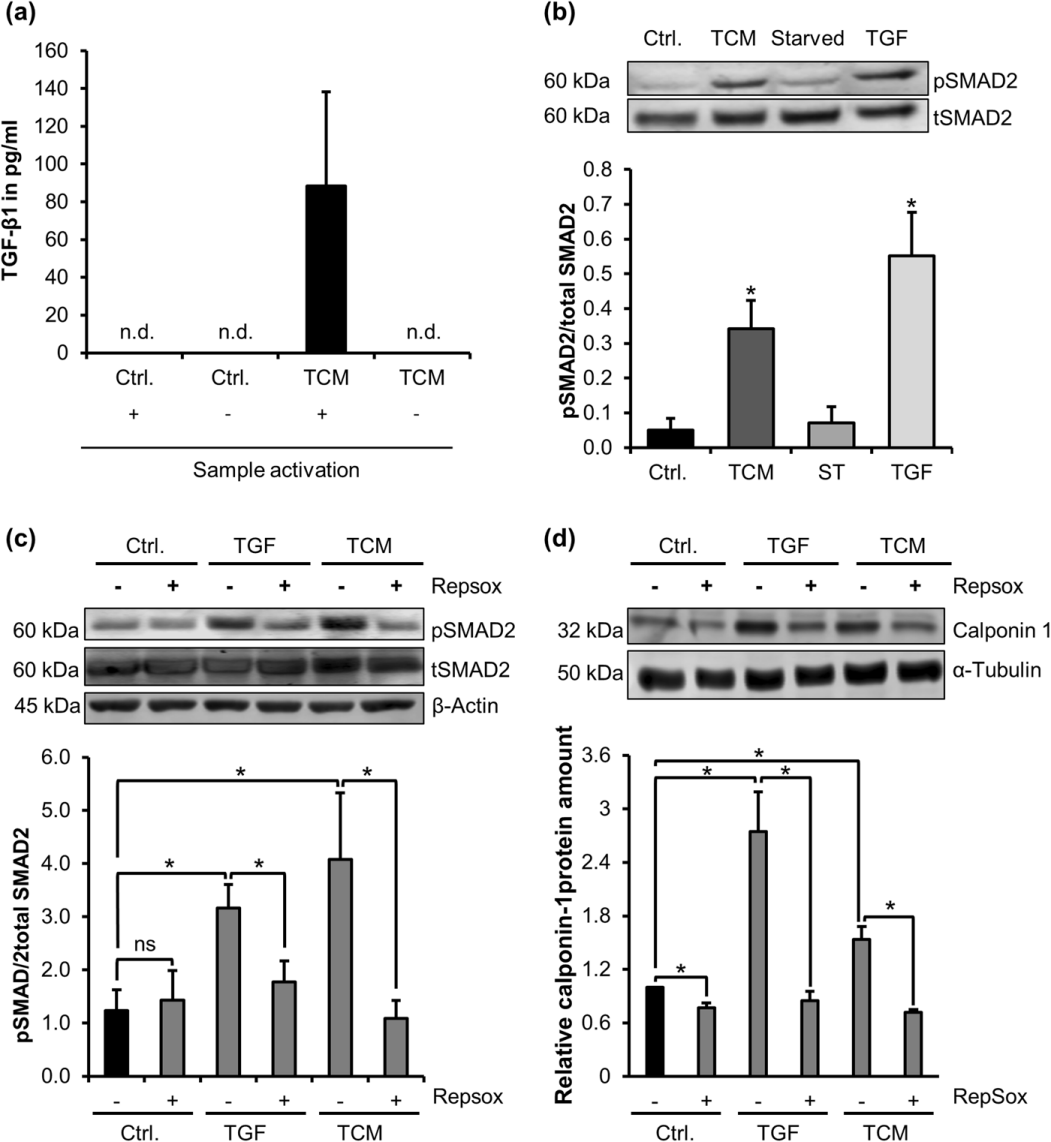
Tumour cell conditioned medium induces TGF-β1-like signalling in MSC. **(a)** TGF-β1 amounts in medium conditioned by HCT8 colorectal carcinoma cells for 72 h (TCM), and identically treated control medium (Ctrl.). The + and – signs below the bar chart indicate whether samples were acid-treated for the release of active TGF-β1. Human recombinant TGF-β1 was used for calibration. *n*.*d*., not detectable. Error bars correspond to SEM (n = 4). **(b)** Western Blot of phosphorylated SMAD2 (pSMAD2) and total SMAD2 (tSMAD2). Cells were treated with TCM or TGF-β1 (TGF) for 1 h, respectively. For quantification, pSMAD2 was normalized to total SMAD2 signal. **(c)** Western Blot of SMAD2 phosphorylation upon TGF-β1 and TCM stimulation after pre-treatment with 200 nM Repsox for 1 h. **(d)** Calponin 1 induction in MSC by 48 h of TGF or TCM stimulation in the presence of RepSox. Quantification normalized to tubulin. Error bars correspond to SD (n = 3). Asterisks indicate significant differences (*p ≤ 0.05) compared to the control sample (Ctrl) according to an unpaired Student’s t test.

The above data suggest that factors secreted by HCT8 colorectal carcinoma cells trigger MSC differentiation in the same manner as TGF-β1 does. We thus tested whether HCT8 conditioned medium contains TGF-β1 and/or elicits TGF-β specific signalling responses. Around 80 pg/ml TGF-β1 was detected in medium conditioned for 72 h by 1 × 10^5^ HCT8 cells/ml (Fig. 3a). TGF-β1 is known to be inactive when complexed with the latency–associated peptide^22^. For ELISA measurements, mature TGF-β1 was released by a sample activation step. In control samples without HCT8 cells and in samples lacking activation, mature TGF-β1 was not detectable.

TGF-β specific signalling events were monitored by analysing SMAD2 phosphorylation after TCM treatment. TCM as well as recombinant human TGF-β1 significantly induced SMAD2 phosphorylation in MSC whilst SMAD2 protein levels remained unaffected (Fig. 3b). In contrast, control media showed no effect on SMAD2 phosphorylation. To further investigate the role of TGF- β1 signalling, cells were pre-treated with the Alk5 kinase inhibitor RepSox. RepSox inhibited SMAD2 phosphorylation by TGF-β1 and TCM treatment (Fig. 3c). Moreover, we could show an impairment of calponin-1 induction by TGF-β1 and TCM upon RepSox treatment (Fig. 3d). Together, these results demonstrate that factors like TGF-β1 are secreted by HCT8 tumour cells and that calponin 1 induction requires Alk5 S/T kinase activity.

### MRTF-A is implicated in inducible MSC differentiation

**Figure 4:**
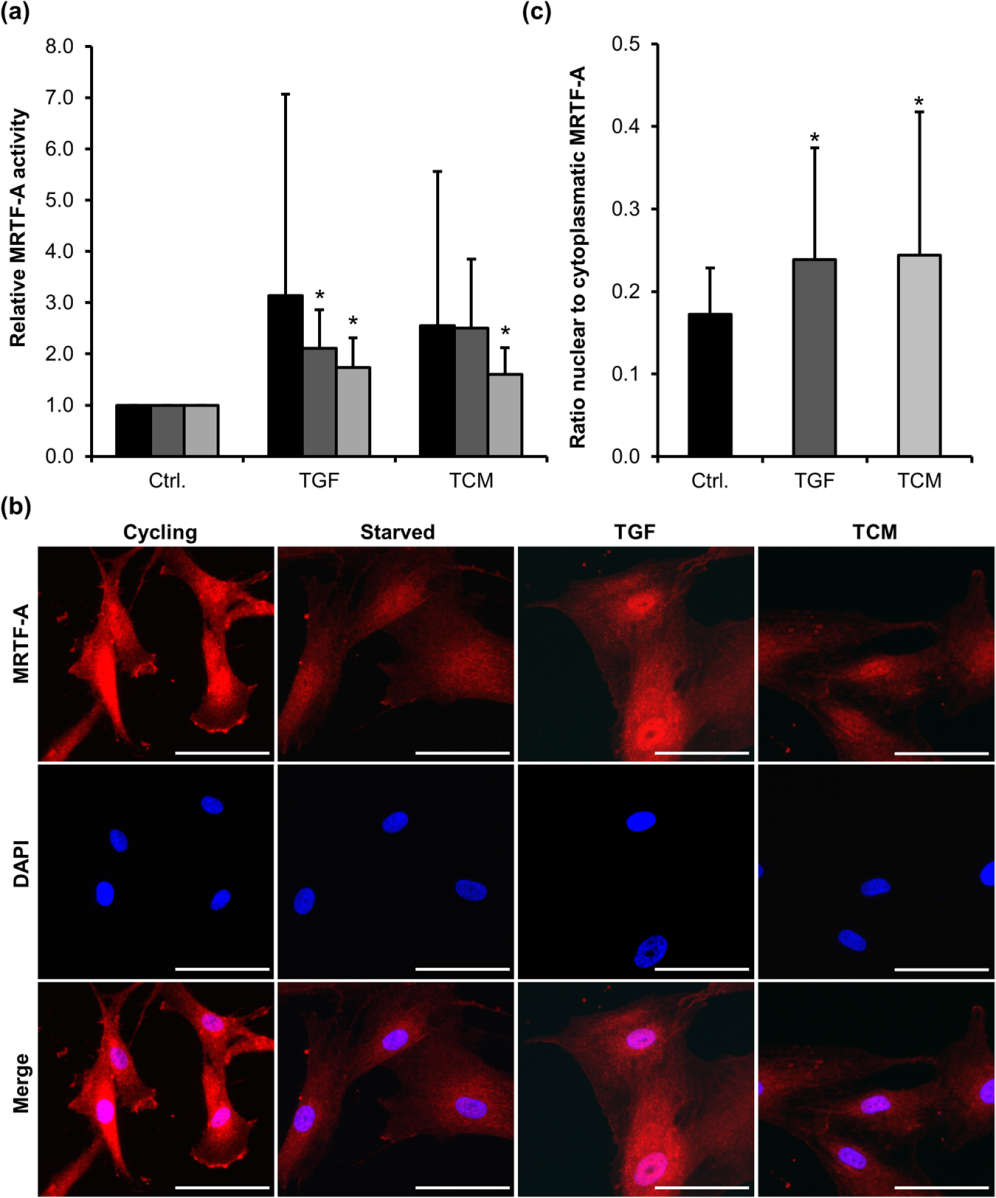
Role of MRTF-A activity during myofibroblastic MSC differentiation. **(a)** MSC stably infected with lentiviral MRTF-SRF dependent luciferase reporter constructs were analysed during MSC differentiation induced by TCM or TGF-β1 (TGF) for the indicated time points (7 h, 24 h, 48 h). Relative luciferase activities normalized to constitutive Crimson Red expression are displayed. Error bars correspond to SD (n = 5). **(b)** Intracellular localization of MRTF-A. Cells were immunostained with anti-MRTF-A antibodies and DNA counterstained with DAPI following 48 h of treatment with TGF-β1 (TGF) or TCM. 20 x magnification, scale bars 20 µm. **(c)** Quantification of MRTF-A displaying the change of nuclear to cytoplasmatic MRTF-A signal in 30 cells (each) from three independent MSC donors. Asterisks indicate significant differences (*p ≤ 0.05) according to an unpaired Student’s t test.

The transcriptional regulator MRTF-A has been implicated in myofibroblastic differentiation^23^. To analyse whether MRTF-A is involved in myofibroblastic differentiation of MSC, we performed luciferase reporter assays using a MRTF-SRF dependent promoter. In lentivirally transduced MSC, we could show an increase in MRTF-A activity after TCM and TGF-β1 treatment (Fig. 4a).

MRTF activation is often associated with its nuclear translocation or retention^10,24^. We thus investigated the subcellular localization of MRTF-A. Immunofluorescence studies indicated slightly increased amounts of nuclear MRTF-A after TCM or TGF-β1 stimulation compared to the control (Fig. 4b). However, in cycling and serum starved cells the MRTF-A signal was not excluded from the nucleus as previously reported for immortalized fibroblasts^10^, but evenly distributed over the cell including cytoplasm and nucleus. Quantification of the nuclear MRTF-A staining, however, revealed that TCM or TGF-β1 stimulation slightly increased the relative nuclear amounts of MRTF-A (Fig. 4c). The data suggest that our primary MSC harbour a substantial proportion of MRTF-A constitutively in the nucleus, which nevertheless is still amenable to signalling (see discussion).

### Impaired myofibroblastic marker expression by MRTF-A RNAi

**Figure 5:**
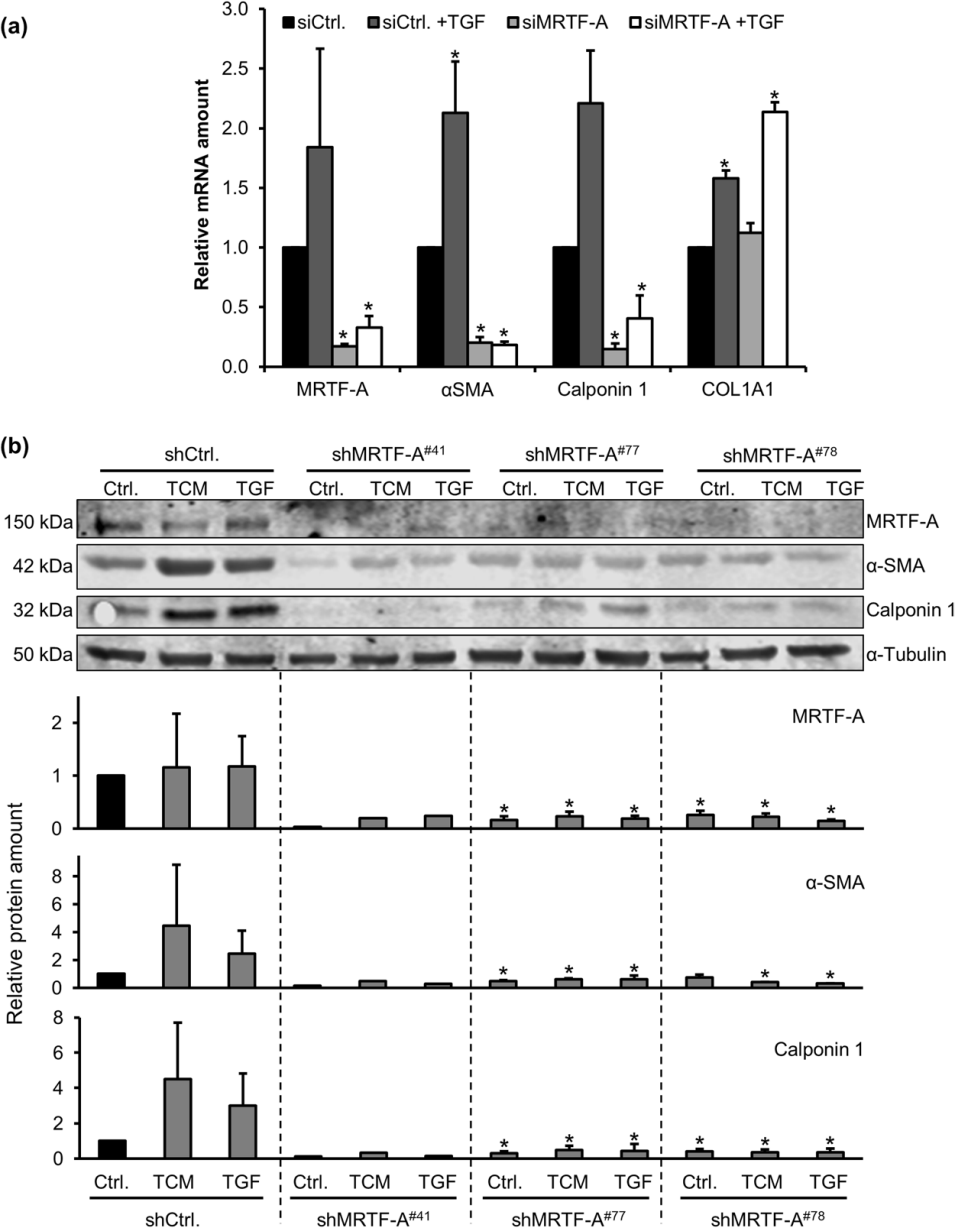
Impaired MSC differentiation by MRTF-A knockdown. (**a)** Expression of MRTF-A and myofibroblastic marker genes upon TGF-β1 treatment (24 h) following transient knockdown by MRTF-A siRNA (siMRTF-A) or control siRNA (siCtrl.). The relative mRNA amounts were determined by quantitative RT-PCR, normalized to the starved siCtrl. Error bars correspond to SEM (n = 4). Asterisks indicate significant differences (*p ≤ 0.05) according to an unpaired Student’s t test. **(b)** Western blot of MRTF-A, α-SMA and calponin 1 in lentivirally transduced MRTF-A knockdown MSC of three individual donors. Control infected cells (shCtrl.) as well as MRTF-A knockdown cells obtained by three independent shRNA constructs (#41, #77, #78) were treated with TCM or TGF-β1 (TGF) for 48 h. The relative protein amounts were normalized to α-tubulin signals. For quantification, data were normalized to the value for the starved shCtrl., which was set to 1. Shown are averages of 3 individual donor MSC. Error bars correspond to SD (n = 3). Asterisks indicate significant differences (*p ≤ 0.05) according to a one-way ANOVA with Dunnett’s multi comparison test (post-hoc).

To test whether MRTF-A is required in the differentiation process, transient siRNA mediated MRTF-A knockdown was combined with TGF-β1 treatment of MSC. Control transfected TGF-β1 stimulated cells showed the same induction pattern of CAF marker genes (α-SMA, calponin 1, COL1A1) as wildtype cells. However, MRTF-A knockdown resulted in a strong and significant reduction of α-SMA and calponin 1 expression, respectively (Fig. 5a). Moreover, the TGF-β1-induced upregulation of α-SMA and calponin 1 was effectively blocked by MRTF-A knockdown. In contrast, expression of COL1A1 was not diminished upon MRTF-A knockdown, neither on its basal, nor on its TGF-β1 induced mRNA levels. These results suggest that TGF-β1 induced expression of the myofibroblastic marker genes α-SMA and calponin 1 requires MRTF-A, while COL1A1 expression is regulated independently of MRTF-A in MSC.

By lentiviral transduction, we next generated stable knockdown MSC. Three different shRNA sequences against MRTF-A (indicated by #41, #77 and #78) were applied and effects compared to a non-targeting control shRNA (shCtrl.) To reduce donor-specific effects in this primary cell model, MSC of three different donors were transduced. The results show a significant downregulation of basal α-SMA and calponin 1 protein amount after MRTF-A knockdown compared to controls (Fig. 5b). Whilst stimulating control MSC with TCM or TGF-β1 lead to an increase in α-SMA and calponin 1 protein signal in controls, this induction was lost in MRTF-A depleted samples. MRTF-A knockdown was validated for all three shMRTF-A constructs used. Together, this demonstrates that MRTF-A plays an important role during myofibroblastic differentiation of MSC by regulating key elements of the myofibroblastic differentiation machinery.

### Tumour-supporting role of MSC towards HCT8 xenografts is reduced upon MRTF-A knockdown

**Figure 6:**
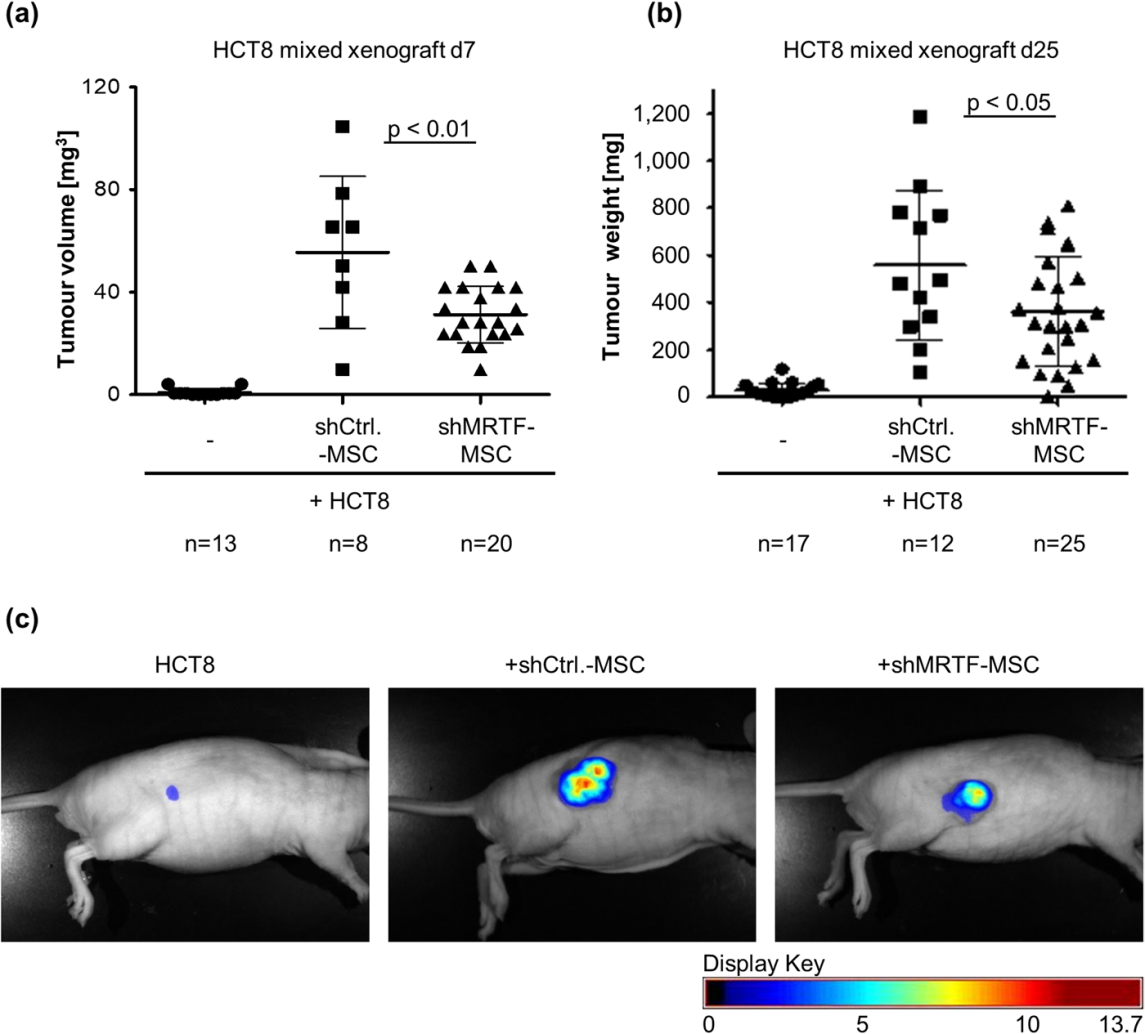
MRTF-A knockdown partially impairs tumour-promoting effects of MSC on HCT8 xenograft growth in mice. 3 × 10^6^ HCT8 cells were coinjected with or without 7.5 × 10^5^ MSC s.c. in athymic nude mice. **(a)** Tumour volumes were determined using calliper measurement at day 7 (d7). **(b)** Mice were killed and the tumours were extracted and weighted after 25 days (d25). One-way ANOVA with Tukey’s multiple comparison test (post-hoc). **(c)** *In vivo* acquisition of multispectral images of DsRed-expressing tumours in athymic nude mice on d25. Pictures were taken via 2.2 CRi Maestro *in vivo* fluorescence imaging system (CRi, Woburn, MA, USA) with Maestro software (2.22). Exemplary grayscale images were overlayed with respective fluorescence images (intensity weighted pseudocolor mode, scale bar displayed beneath).

To elucidate the effects of MRTF-A knockdown on the tumour-supporting activity of MSC and thus on their functional CAF-activity, we performed mixed xenograft experiments. DsRed-labelled HCT8 cells were injected subcutaneously with either shCtrl or shMRTF-A MSC in a ratio of 5:1. After 7 days, HCT8 cells alone formed only tiny tumours (0.87 ± 1.49 mm^3^, mean volume ± SD) (Fig. 6a). When mixed with shCtrl-MSC the mean tumour volume was increased to 55.54 ± 29.73 mm^3^. In comparison, xenografts consisting of shMRTF-MSC and HCT8 cells showed a significant (p˂0.01) reduction in tumour volume (31.27 ± 10.98 mm^3^) compared to the shCtrl-MSC. These data indicate an impaired early tumour growth in the presence of MRTF-A-depleted MSC.

A comparable impact of MRTF-A was shown on the tumour weight after 25 days of xenograft growth (Fig. 6b and Suppl. Fig. S2). The tumour mass increased from 28.3 ± 31.5 mg (average mass ± SD) in HCT8 single tumours to 557.3 ± 316.6 mg in shCtrl-MSC containing control xenografts. Compared to controls, a significant reduction (p˂0.05) in tumour mass (359.7 ± 231.7 mg) was measured in xenografts containing shMRTF-MSC. Moreover, imaging DsRed-fluorescence of the labelled HCT8 allowed visualizing the tumour growth in anaesthetized mice (Fig. 6c). Since the fluorescence signal directly corresponds to the tumour cell number^25^ the result demonstrates that the decreased tumour size was caused by reduced HCT8 cell proliferation and not by failed stroma generation. Consistently, no obvious difference in collagen deposition was observable (Suppl. Fig. S3). Taken together, the results show that MRTF-A controls not only the myofibroblastic differentiation of MSC but also their functional differentiation towards a tumour-promoting CAF phenotype *in vivo*.

## Discussion

The importance of the stroma for tumour growth and progression is increasingly appreciated. A role of CAF is to provide a niche for growing or disseminating cancer cells. Our work demonstrates that the MRTF-A transcription factor is critically involved in the differentiation and tumour supporting function of MSC. The TGF-β1 induced MRTF-A-dependent gene expression in MSC correlated with a MRTF-A dependent growth support of xenografted colorectal carcinoma cells *in vivo*. In the tissue context of the mixed xenografts, however, other signalling events evoked by unidentified secreted factors and cell-cell contacts cannot be excluded to additionally support the MSC function. Yet we could show that MRTF-A is critical in MSC for the emergence of the two main CAF characteristics: the myofibroblastic differentiation as well as the tumour-promoting activity.

The ubiquitous presence of MSC as pericytes as well as their demonstrated recruitment to tumours makes them ideal candidates for CAF^16,26,27^. MSC are therefore not just a model for the investigation of CAF characteristics but are likely progenitors of CAF. Their presence in many tissues, their perivascular localization and their life long persistence in the adult organism argues that MSC and MSC-derived CAF provide a pre-metastatic niche for tumour cells. Consistent with this perspective, our previous work indicated particularly an early support of tumour growth in the presence of MSC, suggesting the provision of a beneficial microenvironment^9^. Expanding this observation, we propose here that MRTF-A is functionally implicated in establishing this pre-metastatic niche by MSC.

Calponin 1, α-SMA and collagens are known markers for myofibroblast differentiation and are upregulated in CAF^18,28,29^. Moreover, their promoters are directly regulated by MRTF transcription factors and respond to perturbations of the actin treadmilling in genome-wide expression studies^13,14^. Indeed, α-SMA and calponin 1 induction depends on MRTF-A during MSC differentiation (Fig. 5), in line with other studies^21,30^. Surprisingly, however, collagen 1A1 expression was found to be independent of MRTF-A. A previous study also described that MRTFs are not required for COL1A1 induction in immortalized murine bone marrow MSC stimulated with TGF-β1^30^. This suggests that in bone marrow derived MSC collagen 1A1 expression is sufficiently controlled by alternative mechanisms. In contrast, MRTF-A is required in heart myofibroblasts for expression of all three collagen 1 genes, whose promoters are directly bound and activated by MRTFs^17,31,32^.

Our data demonstrate that MRTF-A depletion in MSC significantly reduces the mixed HCT8-xenograft growth, but does not abolish it. We showed recently that collagen synthesis plays an important role for the tumour supporting function of MSC towards HCT8 xenograft growth^9^. The lack of obvious changes in collagen expression upon MRTF-A knockdown may contribute to the partially maintained support of xenograft growth (Fig. 5a and Suppl. Fig. 3). This suggests that other pathways or factors are cooperating with MRTF-A for full differentiation into CAF. Candidates may include other myocardin family members, such as MRTF-B and myocardin itself, which regulate similar target genes and are functionally largely redundant. Another putatively involved signalling cascade is the Hippo-YAP pathway: it was recently shown that HPV-E6-immortalised CAF isolated from the murine MMTV-PyMT model require YAP-dependent gene expression for mechanical tissue stiffening, invasion and angiogenesis of tumours^15^. Although YAP-TEAD and MRTF-SRF signalling depend on each other via cytoskeletal remodelling^21^, it remains to be investigated how “YAP-only” targets affect CAF function of MSC upon partial depletion of MRTF-A.

We used TGF-β1 to activate MSC and trigger myofibroblastic differentiation *in vitro*. Tissue associated CAF have been reported to resemble such activated fibroblasts, and TGF-β1 is frequently used as a surrogate stimulus to generate CAF-like cells. However, the precise signals activating coinjected MSC inside the HCT8-xenograft are unknown. We were able to trigger MSC differentiation into myofibroblasts also by HCT8-conditioned medium *in vitro* which suggests the involvement of tumour cell secreted factors. Moreover, we measured considerable amounts of TGF-β1 in TCM and showed that TGF-β1-specific signalling events are triggered by TCM, including a requirement of the Alk5 receptor S/T kinase activity (Fig. 3). A potential model for tumour support *in vivo* therefore involves secretion of TGF-like factors by tumour cells, which attract and activate MSC to become CAF. In turn, the MSC may establish an autocrine TGF-β1 signalling loop, and affect the tumour cells in a paracrine fashion. In support of this model, we found increased TGF-β1 mRNA in MSC undergoing myofibroblastic differentiation (Suppl. Fig. S4).

Endogenous MRTF-SRF transcriptional activity is upregulated in MSC stimulated with either TGF-β1 or TCM (Fig. 4). How TGF-β1 affects actin-MRTF signalling is mechanistically not fully understood, but the MRTF-A- and TGF-β1-induced gene expression signatures significantly overlap^14^. In murine MSC, MRTF-A-dependent α-SMA induction requires RhoA and Cdc42, suggesting a proximal cross-activation of Rho-actin-MRTF-A signalling^30^. Given our experimental conditions, we however noted that in resting cells, MRTF-A was not excluded from the nucleus, nor was α-SMA expression absent. In contrast, Foster *et al*. showed that normal murine fibroblast predominantly exhibited cytoplasmic MRTF-A, whilst stiffer substrates allow MRTF-A accumulation in the nucleus^21^. We speculate that our *in vitro* data overestimated the basal MRTF-A activity, nuclear MRTF-A localization, and basal target gene expression in MSC due to their cultivation on non-physiologically stiff plastic. Nevertheless, MRTF-A is both activated and required during myofibroblastic MSC differentiation under these conditions.

In conclusion, MRTF-A contributes substantially to the stromal support of tumour formation. Given the known MRTF-SRF target genes, it is likely that MRTF-A is implicated in providing a niche and supporting stroma for tumour cells. Enhanced actomyosin contractility, modulation of the ECM-integrin connection, the resulting ECM remodelling, and possibly the secretion of accessory factors may contribute to setting the stage for colonization by cancer cells.

## Material and Methods

### Cell culture

Isolation and cultivation of MSC from human bone marrow (BM) were performed as described previously^33^ with some modifications. All donors had given written informed consent to the additional BM aspiration according to a protocol approved by the institutional Review Board (Ethics Committee of the Medical Faculty of Martin Luther University Halle-Wittenberg; Prüfplan Vers. 2, Amendment 1, Ethikvotum 20.04.2010). We hereby confirm, that all research and methods were performed in accordance with the relevant guidelines and regulations. BM samples aspirated from the iliac crest were diluted with PBS, overlayed on Pancoll human (PAN-Biotech GmbH, Aidenbach, Germany) using SepMateTM-50 tubes (STEMCELL Technologies, Vancouver, Canada) and centrifuged 10 min at 1,000 g. The interface containing mononuclear cells was harvested and washed twice with PBS. After centrifugation at 300 g cells underwent a red blood cell lysis (c.c.pro GmbH, Oberdorla, Germany) followed by PBS wash. Finally, the cells were plated in growth medium on cell culture dishes. Growth medium (DMEM, low glucose, Gibco, Darmstadt, Germany) was supplemented with 10% pooled human platelet lysate (hPL, Transfusion Medicine, University Hospital Halle (Saale)), 1 I.U./ml heparin (ratiopharm GmbH, Ulm, Germany) and 1% antibiotic-antimycotic (Thermo Fisher Scientific, Schwerte, Germany). All experiments were performed with MSC from three different donors, with passage numbers between 3 and 6.

The human CRC cell line HCT8 (ATCC CCL-244) was cultivated in RPMI-1640 (Gibco) containing 10% fetal calf serum (FCS, Gibco) and 1% antibiotic-antimycotic. HEK293T cells (ATCC CRL-3216) were cultivated in DMEM (high glucose, Gibco) supplemented with 10% FCS, 2 mM L-Glutamine (Gibco) and 1% antibiotic-antimycotic. Cells were cultivated in a humidified incubator at 37 °C and 5% CO_2_ in monolayers.

### Generation of tumour conditioned medium, TGF-β1 ELISA and MSC treatment

For the generation of tumour cell conditioned medium (TCM) 10^5^ HCT8 cells/ml were seeded in a 145 mm cell culture dish in 25 ml of low glucose DMEM supplemented with 0.5% BSA (Carl Roth, Karlsruhe, Germany) and 1% antibiotic-antimycotic (DMEM-BSA, starving medium). To generate the respective control medium (Ctrl.) DMEM-BSA without HCT8 cells was handled identically. After 72 h TCM and Ctrl. were harvested, centrifuged (5200 g, 10 min) and activated by acidification to release mature TGF-β1 from its latent complex^34^. In brief TCM and Ctrl. were acidified with 1 M HCl to pH 2, incubated for 1 h at room temperature and neutralized to pH 7.4 with 1 N NaOH. Human TGF-β1 DuoSet ELISA (DY240-05, substrate reagent DY999, R&D Systems, Minneapolis, United States) was performed according to the manufacturer’s instructions to determine amounts of active TGF-β1 in TCM.

For TCM or TGF-β1 treatment, MSC have been seeded with a density of 5000 cells/cm^2^ in a 100 mm dish and grown over night in hPL medium. In general, MSC were washed with PBS prior to treatment and incubated for 24 h or 48 h with TCM, TGF-β1 or Ctrl. TGF-β treatment was performed in 6 ml DMEM-BSA (starving medium) with a final concentration of 10 ng/ml recombinant human TGF-β1 (Promokine, Heidelberg, Germany). Control treatment was carried out using identical medium lacking TGF-β1. The Alk5 inhibitor RepSox (Selleckchem, S7223) was used for pre-treatment at 200 nM for 1 h. Afterwards medium was changed to stimulation or control medium, supplemented with 200 nM RepSox.

### Transient siRNA-based knockdown

Transient small interfering RNA (siRNA) transfection of MSC was performed using Viromer Blue (Lipocalyx, Halle, Germany) according to manufacturer’s instructions in low glucose DMEM supplemented with 10% FCS (DMEM-FCS) and a final concentration of 2 nM siRNA. Cells were harvested after 48 h for subsequent qRT-PCR analysis. Combined siRNA transfection and TGF-β1 treatment was performed as follows: Transfection with a final concentration of 10 nM siRNA overnight was followed by medium change to either TGF-β1 containing or control medium (both starving medium) for 24 h. The siRNA pool containing 30 selected siRNAs against human MRTF-A (NCBI Gene ID: 57591) as well as the negative control were purchased from siTOOLs Biotech GmbH (Planegg/Martinsried, Germany).

### Lentiviral vector production and transduction of cells

To establish stable MRTF-A knockdown in MSC, four commercial lentiviral shRNA vectors (Merck KGaA, Darmstadt, Germany) were used (SHCLND-NM_020831, clone ID TRCN0002999-77, -78, TRCN0000303-841, -842) and compared to the non-mammalian shRNA control plasmid (SHC202). MSC harbouring MRTF-A reporter construct (3DA.Luc-MSC) were generated using the pLVX-shRNA2-Crimson-puro lentiviral vector^35^ exchanging the U6 promoter with the 3D.A-Luc firefly luciferase reporter^36,37^ via ClaI/BstBI. Lentiviral particles were generated by transient transfection of HEK293T cells with polyethylenimine (1 µg/µl, Sigma Aldrich) as previously described^38^. The lentivirus packaging plasmid psPAX2 and the pMD2.G envelope plasmid containing VSV-G were kindly provided by Didier Trono (Addgene plasmid #12259, #12260). After 48 h, lentivirus containing supernatant was harvested and concentrated by ultracentrifugation. For viral transduction, MSC were incubated in DMEM-FCS with 8 μg/ml polybrene (Hexadimethrine bromide, Sigma Aldrich) and 5 × 10^5^ viral particles/µl for 24 h. Transduced MSC were selected with puromycin (0.5 mg/ml, Gibco) for 2 days and constantly cultivated with 0.1 mg/ml puromycin as non-clonal population.

### Immunofluorescence microscopy and quantification

For immunofluorescence microscopy cells were fixed with 3.7% formaldehyde, permeabilized with 0.2% Triton X-100 and blocked with 10% horse serum (Sigma Aldrich, München, Germany), 1% BSA (Carl Roth), 0.05% Triton X-100 (Carl Roth) in PBS. The following antibodies were used: MRTF-A (1:100; #14760, Cell Signaling Technologies, Danvers, United States), α-SMA (1:500; A5228, Sigma Aldrich) and secondary antibodies conjugated with Alexa488 or Alexa546 (1:200; A-11001, A-11010, Thermo Fisher Scientific). DNA was counterstained with DAPI (Sigma Aldrich). Samples were covered with Mowiol 4–88 (Carl Roth) and imaged with an Axio Observer 7 (Zeiss, Jena, Germany) equipped with a monochrome Axiocam.

Quantification was done by corrected total cell fluorescence using ImageJ/Fiji^39^ (CTCF: integrated density – (area of selected cell × mean fluorescence of background readings)). ROI’s defined by DAPI staining were transferred to pictures displaying MRTF-A signal to measure nuclear signal intensities. Whole cell signal was measured by tracing the cells with the selection tool.

### Western blot

Immunoblotting was performed as described previously^33^ with the following antibodies: MRTF-A 1:500, α-SMA 1:500, calponin-1 1:250 (sc-58707, Santa Cruz Biotechnology, Dallas, United States), α-tubulin 1:1000 (T9026, Merck), Smad2 1:1000 (#3122, Cell Signaling Technologies), phospho-Smad2 1:1000 (#3104, Cell Signaling Technologies), fluorophore-labelled secondary antibodies (dilution 1:15000; 926–68071, -68070, -32210; Li-Cor, Lincoln, United States) and HRP-conjugated anti-rabbit antibody (1:1000; #7076, Cell Signaling Technologies). HRP signal was detected after incubation with chemifluorescent substrate (P/N 928–30005, Li-Cor). Detection and quantification of the fluorescence signals was performed with an Odyssey CLx system (Li-Cor) using the associated software. For collagen quantitation, cells were seeded in 6 well plates and stained with Sirius Red according to a published protocol^40^. Relative collagen deposition was calculated by normalisation for cell density by crystal violet.

### RNA isolation, cDNA synthesis, qRT-PCR

Total RNA was isolated using the NucleoSpin RNA XS kit (Macherey-Nagel, Düren, Germany) and cDNA synthesis from 500 ng RNA was performed in 20 µl reaction volume using anchored Oligo dT primers within the Verso cDNA synthesis kit (Thermo Fisher Scientific) according to manufacturer’s instructions. Endogenous target gene expression by qRT-PCR was analysed with the LightCycler 480 II instrument (Roche) and a DyNAmo ColorFlash SYBR green qPCR kit (Thermo Fisher Scientific), using the following primers: MRTFA (forward: 5′-GAGCCAGACTAGCCGATGAC-3′, reverse: 5′-CACAGAACCCTGGGACTCAT-3′), ACTA2 (forward: 5′-CGGTGCTGTCTCTCTATGCC-3′, reverse: 5′-AGCAGTAGTAACGAAGGAATAGCCA-3′), CNN1 (forward: 5′-CTGTCAGCCGAGGTTAAGAAC-3′, reverse: 5′-GAGGCCGTCCATGAAGTTGTT-3′), COL1A1 (forward: 5′-CGATGGATTCCAGTTCGAGTAT-3′, reverse: 5′-GACAGTGACGCTGTAGGTGAAG-3′) and the reference genes ALAS1 (forward: 5′-CTGCAAAGATCTGACCCCTC-3′, reverse: 5′-CCTCATCCACGAAGGTGATT-3′) and GAPDH(forward: 5′-ACCCAGAAGACTGTGGATGG-3′, reverse: 5′-TTCTAGACGGCAGGTCAGGT-3′). Relative gene expression levels were calculated according to the method described by Pfaffl^41^.

### Reporter assays

7 × 10^4^ 3DA.Luc-MSC reporter-MSC were seeded in a 12-well plate and grown overnight. Cells were washed once with PBS and medium changed to starvation medium for 24 h. Cells were stimulated for 7 h, 24 h and 48 h (TCM, TGF-β1 or Ctrl). The luciferase reporter assay was performed using the Dual-Glo luciferase assay kit (Promega, Madison, WI) and the GloMax® 96 luminometer (Promega). Firefly luciferase values were normalized to E2-Crimson fluorescence intensities, measured with CLARIOstar microplate reader (BMG Labtech, Ortenberg, Germany). Results were displayed as fold changes.

### Animal studies

All experiments were performed according to institutional guidelines. The study had been approved by federal authorities (Landesverwaltungsamt Sachsen-Anhalt, Referat 203 Verbraucherschutz, Veterinärangelegenheiten; study no.: 42502-2-1248MLU and 42502-2-1477MLU). All animal experiments were performed with male six- to eight-week-old athymic nude-fox n1 nu/nu mice (Charles River, Sulzfeld, Germany). Mixed xenografts were established by subcutaneous (s.c.) injection of DsRed-labelled HCT8 cells mixed with MSC (either shCtrl or shMRTF-A) into the flanks of nude mice. Per tumour 3 × 10^6^ HCT8 cells were co-injected with 0.75 × 10^6^ MSC in 150 µl PBS without supplements. Xenografts of HCT8 cells without admixture of MSC served as control. Each animal carried two contralateral s.c. xenografts which were randomly distributed, to avoid side specific artefacts. Tumour dimensions were determined using calliper measurement at different time points. Tumour volume was calculated by the modified ellipsoidal formula tumour volume = π/6*length*width² as described previously^42,43^. After 25 days mice were killed and the tumours were extracted and weighted. Every experiment was done at least in triplicates. None of the animals had to be sacrificed prematurely due to tumour burden or impaired vital parameters. A 2.2 CRi Maestro *in vivo* fluorescence imaging system (CRi, Woburn, USA) was used to acquire non-invasive multispectral images of DsRed-expressing tumours as described previously^44^.

### Statistical analyses

Data represent means with corresponding SD including minimum of three independent biological replicates. Statistical analysis was performed using SPSS 24.0 software (SPSS Inc., Chicago, United States) applying an unpaired two sample Student’s t-test, a one-way ANOVA with Dunnett’s multi comparison test (post-hoc) or a one-way ANOVA with Tukey’s multiple comparison test (post-hoc) as indicated. Significance is indicated by *p ≤ 0.05.

## Supplementary information

Supplementary Information

## Data Availability

The datasets generated during and analysed during the current study are available from the corresponding author on reasonable request.
