# Supplementary material for: MRTF-A controls myofibroblastic differentiation of human multipotent stromal cells and their tumour-supporting function in xenograft models
Source: Sci Rep. 2019 Aug 13;9:11725. doi: 10.1038/s41598-019-48142-z (PMC6692381; doi:10.1038/s41598-019-48142-z)
Supplement: Supplementary file 1 — Supplementary Information [file 41598_2019_48142_MOESM1_ESM.pdf]

MRTF-A controls myofibroblastic differentiation of human multipotent stromal cells and their tumour-supporting function in xenograft models

Sara Werner [1,#], Jana Lützkendorf [2,#], Thomas Müller[2], Lutz P. Müller [2,*] and Guido Posern [1,*]

Supplementary Figures

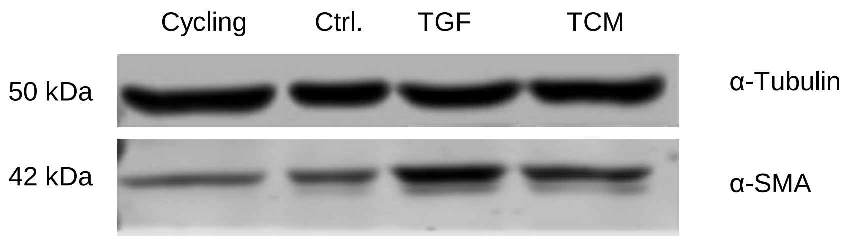

Supplementary figure S1: Comparison between cycling and starved control MSC.

Western blot of α-SMA and α-Tubulin (loading control) in MSC. Cycling steady state cells, starved cells, and cells treated with TGF and TCM are compared for α-SMA protein amount. Treatment was performed for 48 h. One representative Western blot is shown.

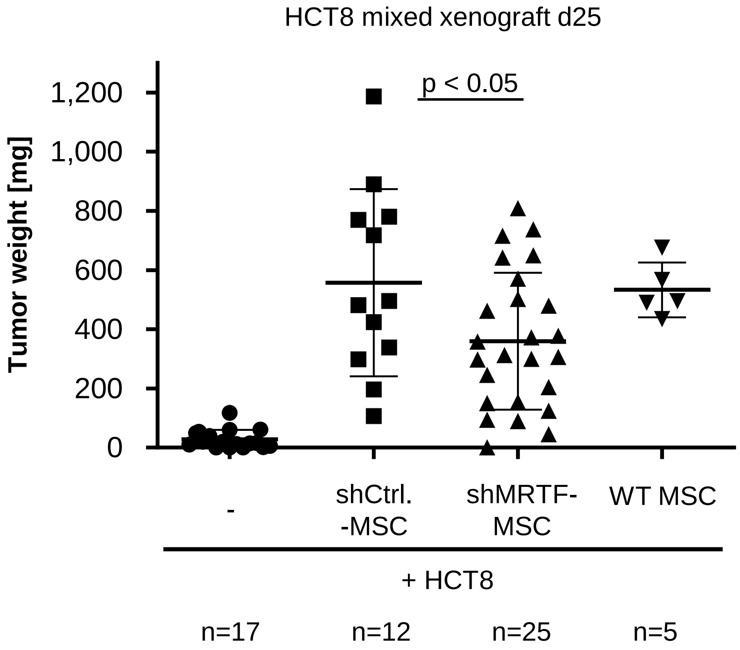

Supplementary figure S2: Extended figure 6 for comparison of wt MSC with shRNA infected MSC.

$3x10^6$ HCT8 cells were coinjected with or without $7.5x10^5$ MSC s.c. in athymic nude mice. Mice were killed and the tumours were extracted and weighted after 25 days (d25). One-way ANOVA with Tukey's multiple comparison test (post-hoc).

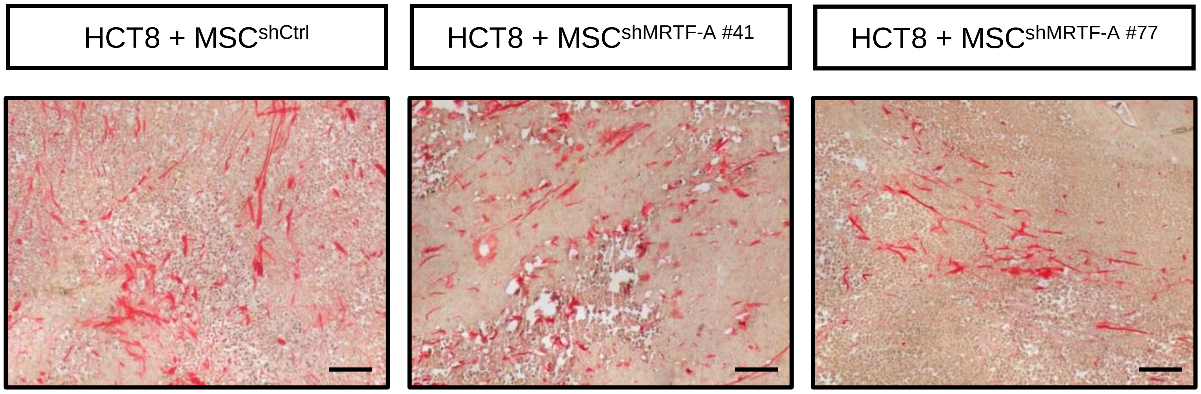

Supplementary figure S3: Sirius Red staining of excised xenograft tumours.

Xenograft tumours were fixed in 5 % formalin, embedded in paraffin and cut into 4 µm slices. The staining was performed as followed: First, paraffin sections are de-waxed and hydrated followed my nuclei staining with Weigert's haematoxylin (Haematoxylin solution A [X906.1] and B [X907.1] by Weigert; Carl Roth) for 8 minutes. Afterwards slides were washed for 10 minutes in running tap water followed by picro-sirius red (Direct Red 80 [Sigma, Cat. #365548] in saturated aqueous solution of picric acid [Sigma; #197378]) staining for 1 h. Next slides were washed twice with acidified water (5 ml acetic acid [glacial] in 1 liter of water). By vigorous shaking most of the water has been removed from the slides. Finally samples have been dehydrated with 100 % ethanol in three changes, cleared in xylen and mounted in resinous medium.

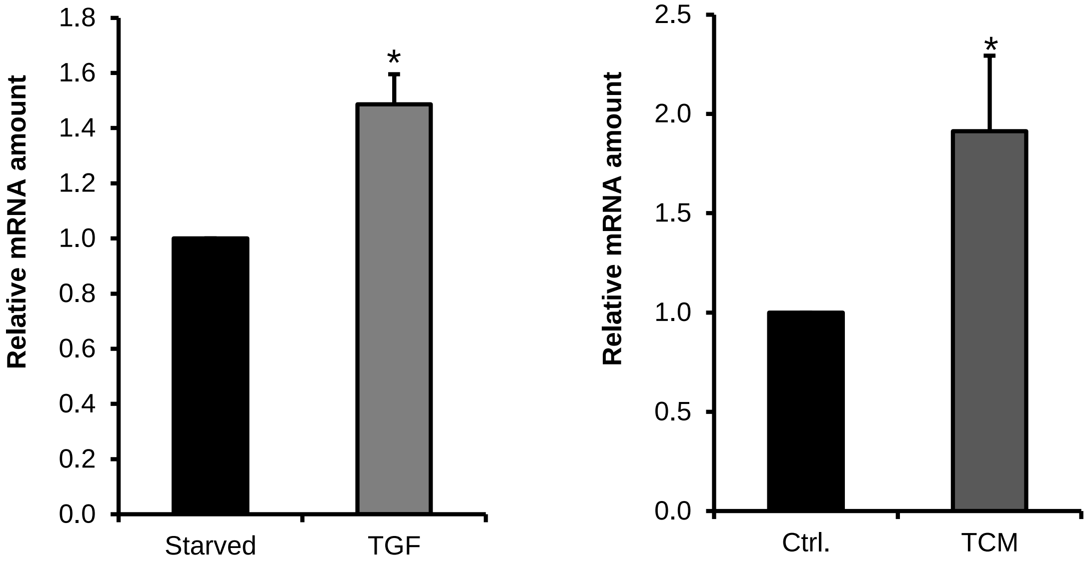

Supplementary figure S4: TGF-β 1 mRNA induction upon TGF-β1 and TCM treatment

MSC were treated with TGF-β1 (left panel) or tumour cell conditioned medium (TCM; right panel) for 24 h. Relative mRNA of TGF-β1 was quantified by real-time RT-PCR and normalized to ALAS and GAPDH. Data were normalized to the value for the starved control or TCM Control medium (Ctrl.) which was set to 1. Error bars correspond to SD (n=3). Asterisks indicate significant differences *$p \leq 0.05$ according to an unpaired Student's t test.

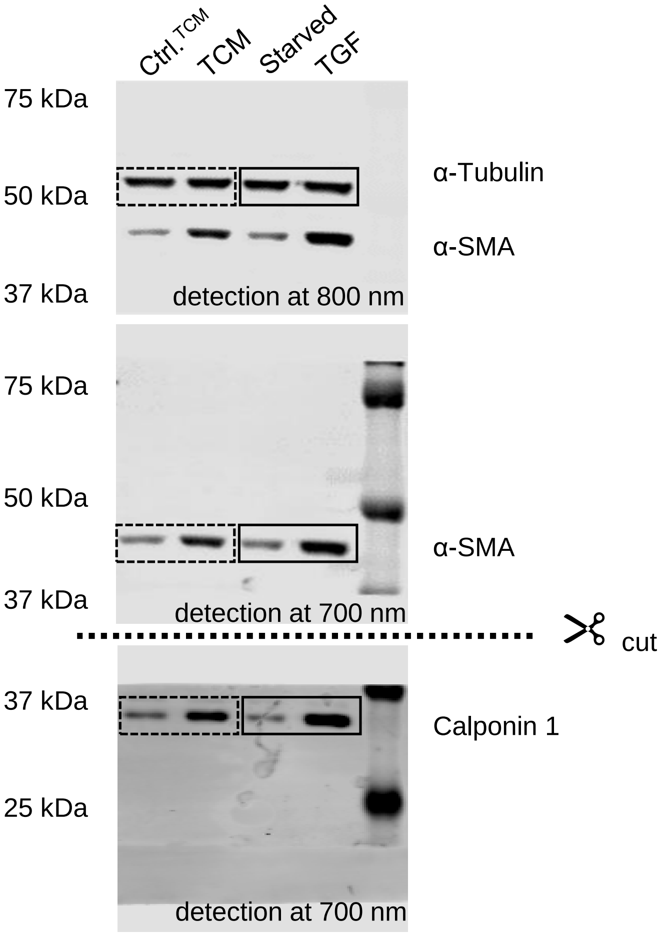

Supplementary figure S5: Original blots regarding the data shown in Fig 1 (b) and (d)

The original blots from MSC which underwent TCM and TGF treatment, displayed in Fig 1 (B) and (D) in the main manuscript, are shown here. Samples were run on the same SDS gel and blotted to the same membrane. The treatment was done for 3 independent MSC batches from different human donors. One MSC batch was chosen to display results representatively. The black boxes tag protein signals shown in Fig 1 (B), whereas dotted boxes mark the signals shown in Fig 1 (D). Since molecular weights of the proteins to be detected are located close to each other, the membrane was horizontally cut into pieces, prior to first antibody incubation. Signal acquisition was done using Li-Cor CLx imaging system. Calponin 1 signal was detected on the lower part of the membrane (lowest picture), the cut dividing the membrane was made in middle of the 37 kDa band of the protein standard (Precision Plus Protein Standard, BIO-RAD). Detection of α-SMA and α-tubulin signals was done on the same piece of membrane using secondary antibodies labelled to different fluorophores. At first α-SMA signal was acquired (middle picture) using IRDye 800CW Goat anti-Mouse IgG secondary antibody (Li-Cor, #926-32210), followed by primary α-tubulin antibody and IRDye 680RD Goat anti-Mouse IgG (Li-Cor, #926-68070) secondary antibody. As the 800CW Goat anti-Mouse did not saturate the mouse IgG, there is a α-SMA and tubulin signal detected using the second secondary antibody IRDye 680RD Goat anti-Mouse (top picture).

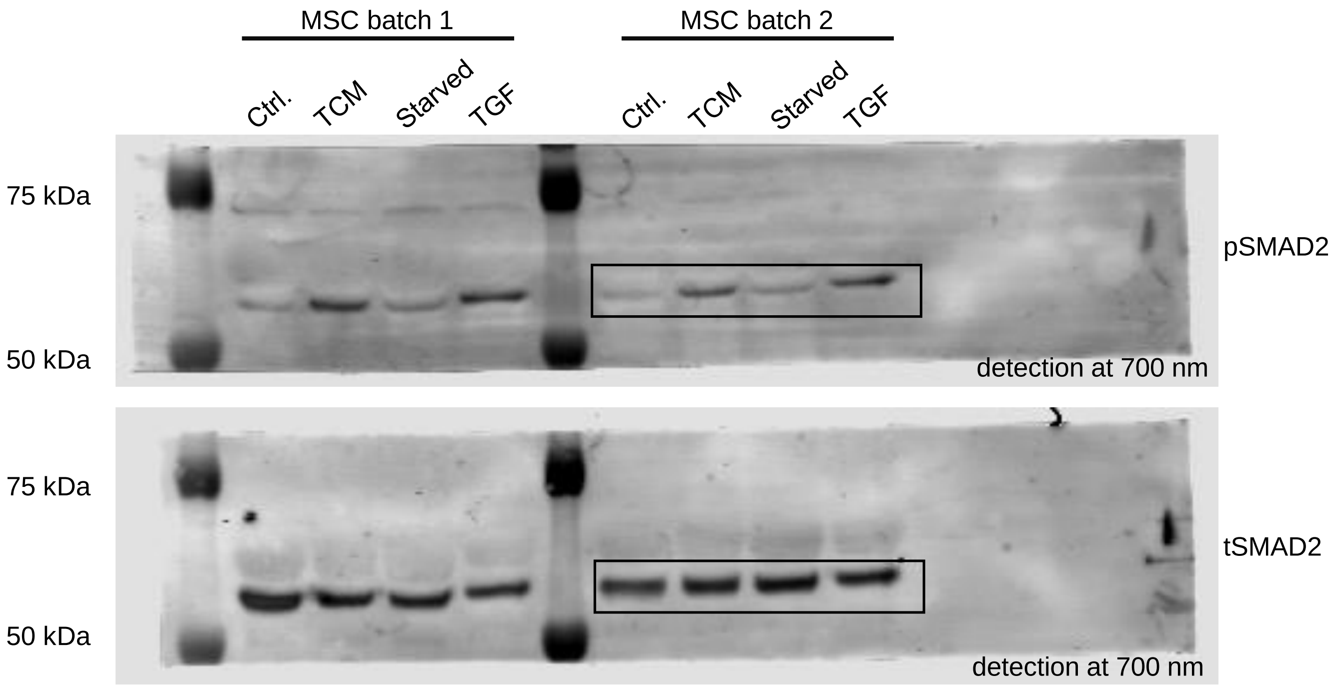

Supplementary figure S6: Original blots regarding the data shown in Fig 3 (B)

The samples shown in Fig. 3 (B) and appropriate originals blots shown here, were run on two separate gels. The upper picture displays the Phosphorylated SMAD2 (pSMAD2) level and the lower part the total SMAD2 (tSMAD2) signal of two independent MSC batches, after TCM, TGF of control treatment for 1h. The sample volume, SDS-PAGE and Western blot settings (buffer, duration etc.) were identical. Gels and blots were run in parallel. pSMAD2 and tSMAD2 signals were detected using chemifluorescent HRP substrate (P/N 928-30005, Li-Cor) and the Li-Cor CLx imaging system. Both primary antibodies used are of the same species by what the use of two gels was unavoidable. The black box marks the sample set representatively displaying the results in the main manuscript.

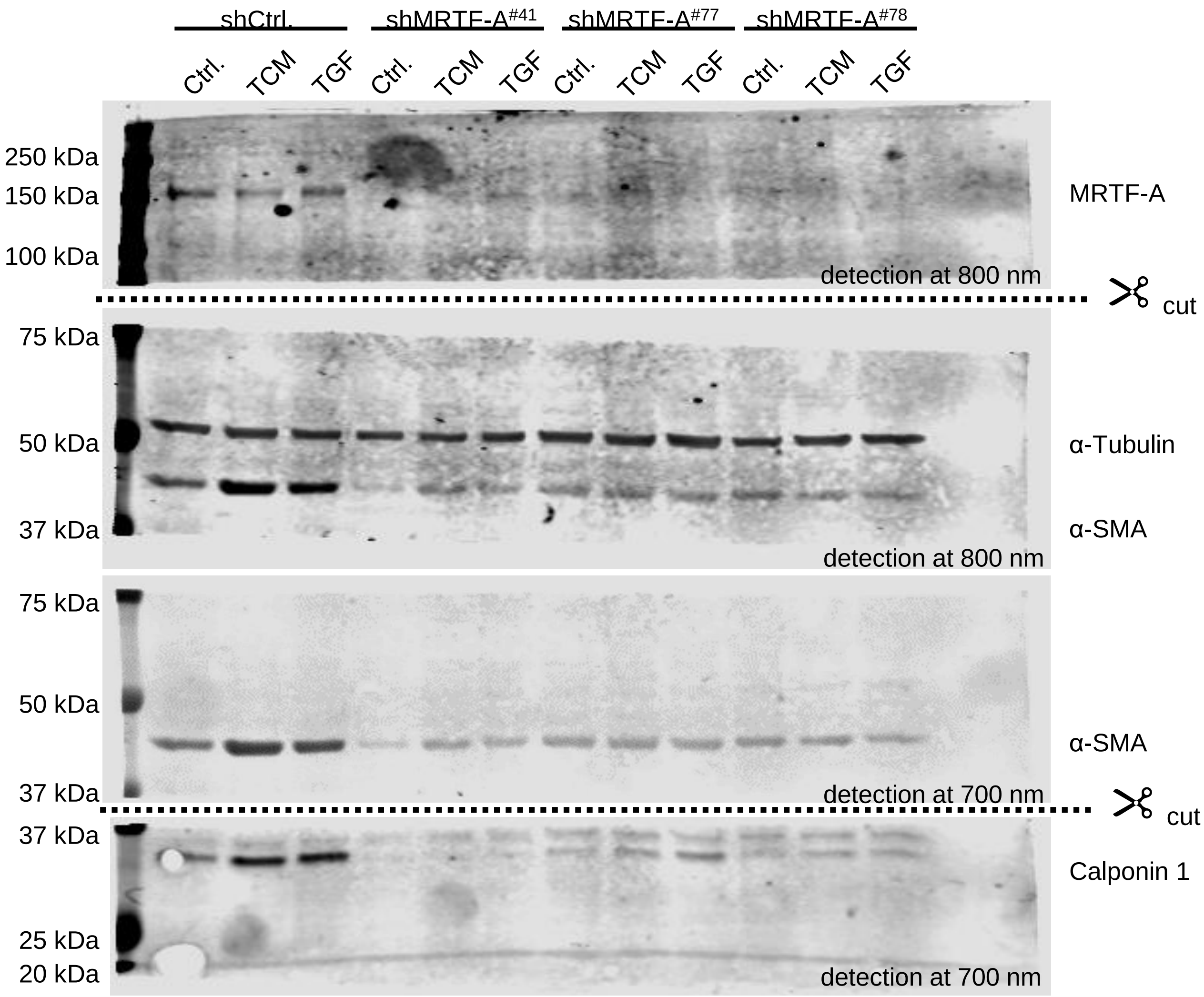

Supplementary figure S7: Original blots regarding the data shown in Fig 5 (B)

The original blots showing samples of MRTF-A knockdown MSC which underwent TCM and TGF treatment, displayed in Fig. 5 (B) in the main manuscript, are shown here. Samples were run on the same SDS gel and blotted to the same membrane. The treatment was done with 3 independent MSC batches from different human donors the shCtrl. and different shRNA constructs (#41, #77, #78). The MSC batch shown here was chosen to display the results representatively in the main manuscript. Since molecular weights of the proteins to be detected are located close to each other, the membrane was horizontally cut into pieces at 37 kDa and 75 kDa band of the protein standard (Precision Plus Protein Standard, BIO-RAD), prior to first antibody incubation. Calponin 1 signal was detected on the lower part of the membrane (lowest picture). Detection of α-SMA and α-tubulin signals was done on the same piece of membrane using secondary antibodies labelled to different fluorophores. At first α-SMA signal was acquired (second lowest picture) using IRDye 800CW Goat anti-Mouse IgG secondary antibody (Li-Cor, #926-32210), followed by primary α-tubulin antibody and IRDye 680RD Goat anti-Mouse IgG (Li-Cor, #926-68070) secondary antibody. As the 800CW Goat anti-Mouse did not saturate the mouse IgG, there is a α-SMA and tubulin signal detected using the second secondary antibody IRDye 680RD Goat anti-Mouse (picture second from the top). The top picture displays the MRTF-A signal.
